# Factors influencing timely diagnosis of autism in China: an application of Andersen’s behavioral model of health services use

**DOI:** 10.1186/s12888-025-06590-0

**Published:** 2025-02-18

**Authors:** Zhuoqing Li, Xuejing Niu, Patrick C. M. Wong, Huanyu Zhang, Li Wang

**Affiliations:** 1 Brain and Mind Institute, The Chinese University of Hong Kong, Shatin, N.T., Hong Kong SAR China; 2 International Office, Guilin University of Aerospace Technology, Guilin, 541004 China; 3 Department of Linguistics and Modern Languages, The Chinese University of Hong Kong, Shatin, N.T., Hong Kong SAR China; 4 Clinical Big Data Research Center, The Seventh Affiliated Hospital, Sun Yat-sen University, Shenzhen, 518107 China; 5 Shenzhen Research Institute, The Chinese University of Hong Kong, Shenzhen, 518000 China

**Keywords:** Autism, Andersen's behavioral model, Timely diagnosis, Influencing factors, China

## Abstract

**Background:**

Timely diagnosis of autism is pivotal for accessing crucial supports and services. However, achieving it remains a persistent challenge, particularly in countries like China where the healthcare system is characterized by its intricate network and often resulting in fragmented care delivery and disparities in access.

**Methods:**

A cross-sectional questionnaire survey was conducted among families with autistic children aged between 1 and 17 years in Chinese Mainland. Andersen’s Behavioral Model guided the assessment of predisposing, enabling, and need factors.

**Results:**

The study revealed that 86.24% of Chinese children did not receive a formal diagnosis until after 24 months, with an average gap of 10.93 months between parents/caregivers’ initial concerns and diagnostic confirmation. Predisposing factors of the child’s current age emerged as a significant predictor for timely diagnosis. Enabling factors, including residence-hospital distance and experience of misdiagnosis were significant. Additionally, the severity level at diagnosis was identified as a predictor for timely diagnosis.

**Conclusions:**

Our findings revealed that predisposing, enabling, and need factors contributed to the complex landscape of autism diagnosis in China. Strategies including implementing routine screening programs and adopting a multidisciplinary approach are crucial for timely identification and diagnosis, particularly for mild and moderate cases. Additionally, there is an urgent need for specialized autism training for healthcare professionals, the development of structured referral systems, and the expansion of telehealth solutions to ensure equitable access to diagnosis services across regions. By addressing these challenges, policymakers and healthcare providers can improve the accessibility and timeliness of autism diagnosis, ultimately enhancing the quality of life for affected individuals and their families.

## Introduction

Autism Spectrum Disorder (ASD, referred to as autism below) is a neurodevelopmental disorder with a typical onset in early childhood. The diagnostic criteria include difficulties in social interaction and repetitive behavior patterns and interests [1]. Autism is a spectrum condition, meaning symptoms and their severity vary widely. For example, some autistic children may have advanced language skills and cognitive abilities, while others may be non-verbal or rely on alternative communication methods such as gestures or assistive technology [2]. Timely diagnosis of autism is essential for accessing early intervention and other services. This leads to enhancements in language and communication skills, social functioning, and coping strategies, while also mitigating possible co-occurring conditions such as disruptive, aggressive, and self-injurious behaviors during development [3, 4]. Additionally, timely diagnosis fosters the creation of an autism-friendly environment, empowering caregivers, educators, and other individuals to better understand the child’s behavior patterns and challenges, thus promoting more supportive and inclusive social networks [5].

Despite suggestions that autism can be identified as early as 18 to 24 months based on distinguishable core symptoms from typical development or other developmental conditions [6], a recent meta-analysis reveals a significant delay in the average global age for autism diagnosis, falling within a range of 30.90 to 74.70 months [7]. Consequently, achieving timely diagnosis remains a persistent challenge in practice. Multiple factors such as sociodemographic and economic differences, health status and healthcare access influence a timely autism diagnosis. Prior research has shown that a timely diagnosis of autism is influenced by factors at the child and family levels. These include characteristics such as the child’s sex and age, the severity of autistic symptoms, any co-occurring intellectual or cognitive disabilities, language delays, and a diagnostic history of other developmental disorders. Additionally, family factors such as household income, parental education levels, marital status, and parents’ concerns about social communication delays also play a role in the timing of the diagnosis [8–16]. Diagnostic delay is also influenced by factors at the healthcare system level such as distance to diagnostic centers, number of providers available and insurance types [12, 13, 15]. Nonetheless, mixed results were found in previous studies. For example, while a US-based study conducted by Kalkbrenner et al. [12] showed that living in an area with a high density of health providers can lead to earlier diagnosis, the French study by Rattaz et al. [17] found no effect of the distance from the residence place to the hospital center. This difference underscores the importance of considering country-specific factors, including healthcare system structures and access to services, which can influence the timing of diagnosis differently across countries. Similarly, studies in the US and Denmark showed that higher levels of household income and parental education may lead to an earlier diagnosis, whereas studies conducted in the UK and France demonstrated converse results and the Israeli study did not find any association between the age of diagnosis and parental education [8, 17–20]. These inconsistencies highlight the need for further research to better understand how factors specific to different countries impact the timing of autism diagnosis. A more comprehensive approach that takes into account these country-specific variations is essential for developing a clearer understanding of the multifactorial influences on diagnosis.

In China, due to the uneven distribution of high-quality health care, people often need to travel a long distance to access appropriate care, especially among those with complex care needs [21]. Furthermore, the challenges surrounding the timely diagnosis of autism are not only structural but also culturally nuanced. There is still significant stigma surrounding autism, and many families hesitate to seek a diagnosis or disclose their child’s condition due to fear of discrimination or social judgment. Additionally, a lack of public awareness about autism makes these challenges even worse. A recent study comparing public autism knowledge and stigma between China and the United States found that only 57–65% of Chinese citizens demonstrated adequate autism knowledge, compared to 86–91% in U.S. citizens [22]. Moreover, 38% of Chinese citizens endorsed stigma toward autism, compared to only 14% in the United States. These cultural factors are compounded by structural barriers such as the scarcity of child psychiatrists and pediatricians, incomplete implementation of early screening programs, and economic disparities between urban and rural areas [23–28]. Addressing these cultural and structural challenges is critical to improving the timeliness and accessibility of autism diagnosis in China.

Several studies have looked into the factors that affect the timely diagnosis of autism in China [13, 29, 30]. However, most of these studies were performed in relatively developed provinces and primarily focused on factors at the individual levels, such as age, sex, socioeconomic status, and parental concerns. To better understand the delays in diagnosis, a broader, more comprehensive approach is needed. Andersen’s Behavioral Model of Health Services Use offers a useful framework for considering not only individual factors but also the healthcare system and societal influences. This model has been widely used to examine patient access to healthcare, especially in the scenarios of delayed diagnosis and patients’ decisions regarding when, where, and how to seek care [31, 32]. As Andersen’s Model suggested, individuals’ use of health care services depends on three key factors: (1) predisposing factors (i.e., characteristics that exist before the onset of an illness), (2) enabling factors (i.e., resources and support mechanisms that facilitate or hinder an individual’s ability to access healthcare services), (3) need factors (i.e., an individual’s perceived or evaluated need for healthcare services). Prior research has emphasized the importance of these three factors varies by the healthcare system and the purpose of the health services [33, 34].

To the best of our knowledge, no studies have utilized Andersen’s Behavioral Model to comprehensively assess the factors associated with the timely diagnosis of autism in China. However, Andersen’s Behavioral Model has been effectively applied in several low- and middle-income countries, providing useful insights into healthcare access and diagnostic delays. For example, a study conducted across six Latin American and Caribbean countries used Andersen’s Behavioral Model to identify determinants of the age of autism diagnosis [15]. The study found that factors such as public health coverage, medical comorbidities, and symptom severity significantly influenced the timing of diagnosis. This study demonstrated the model’s utility in exploring diagnostic delays in contexts with limited healthcare infrastructure, similar to the challenges faced in China, particularly in rural regions. Therefore, this study aims to apply Andersen’s Behavioral Model as a framework and systematically assess factors influencing patient access to a timely diagnosis in China. Based on previous findings and the structure of Andersen’s Model, we hypothesized that timely diagnosis of autism in China may be influenced by (1) predisposing factors including the child’s age and sex, the educational level and marital status of the child’s family, (2) enabling factors including residence location, household registration, economic level of the family, the experience of misdiagnosis and number of clinicians seen before diagnosis, and (3) need factors including the severity of symptoms, initial symptoms and comorbidities of the child.

## Methods

### Data and sample

A cross-sectional questionnaire survey was conducted in Chinese Mainland from March to September 2023. Inclusion criteria required participants to be (1) parents or caregivers of autistic children and (2) residents of Chinese Mainland. A total of 489 parents/caregivers of autistic children aged 1–17 years completed the survey. To ensure data quality, two screening questions were included in the survey. These questions aimed to confirm that participants’ children had been officially diagnosed with autism by a qualified clinician. Based on responses to these screening questions, (1) 26 participants were excluded because they reported that their child had not received an official diagnosis and (2) 78 participants were excluded because they reported a non-autism diagnosis (e.g., communication disorders, attention-deficit/hyperactivity disorder, specific learning disorders, or global developmental delay). In addition, (3) three were excluded because they resided outside Chinese Mainland (including 2 from other countries and 1 from Hong Kong), and (4) four participants were excluded for providing an invalid age of diagnosis (e.g., diagnosed before their birthday). This left a final sample of 378 completed and eligible surveys included in the analysis.

Recruitment efforts were widespread, utilizing various channels to reach participants across diverse provinces in Chinese Mainland. Participants were recruited through social media platforms (e.g., WeChat), parent/caregiver associations, non-profit organizations, and autism intervention institutions. Recruitment materials included digital flyers, social media posts, and direct invitations distributed through these channels. These materials provided a QR code linking to the survey and included information about the study’s purpose, protocol, and voluntary nature of participation. The survey was conducted through Wenjuanxing, a widely accepted online questionnaire survey platform in China for data collection to ensure broad accessibility. Participants accessed the survey by scanning the QR code, which directed them to an explanation of the study’s purpose and protocol. Consent was obtained before they proceed with the survey. Participants were informed that they could withdraw at any point while completing the survey. To ensure participant anonymity, no personal information such as names or phone numbers was collected. Additionally, no monetary compensation was provided for survey completion. The study received ethics approval from the Shenzhen Research Institute of the Chinese University of Hong Kong (PJ-202210B).

### Measures

This study constituted a segment of a comprehensive survey investigating the current circumstances of autistic children and their families in China. The overarching survey covered child and family demographics, diagnostic experiences, intervention encounters and associated financial outlays, parent/caregiver self-efficacy, and quality of life. The survey took approximately 15 to 20 min to complete based on pilot testing, as noted in the protocol shared with participants upon scanning the QR code. For this specific study, we focused on data pertaining to child and family demographics, as well as diagnostic experiences. Employing Andersen’s Behavioral Model for healthcare services access and utilization [15, 34, 35], variables were categorized into predisposing, enabling, and need factors.

Predisposing factors encompass characteristics existing before the onset of an illness, including (1) the age of the autistic child in months, calculated from birth to survey completion; (2) the sex of the child (i.e., female and male); (3) educational level of the parent (raging from no schooling to postgraduate study), with representatives selected based on higher levels of education among parents; (4) marital status of parents.

Enabling factors represent resources and support mechanisms facilitating or hindering healthcare access. Given the prominence of the household registration (Chinese Hukou) system in Chinese socio-economic structures [36], four related variables were examined (1) the household registration location was categorized into tiers (Tier 1 to 5) based on commonly accepted classifications in China [37, 38]. Tier 1 cities, such as Beijing, Shanghai, Guangzhou, and Shenzhen, represent the most developed, with high GDP, advanced infrastructure, and robust healthcare systems. Emerging “new Tier 1 cities” like Chengdu, Hangzhou, and Wuhan are rapidly developing, with significant growth in infrastructure and healthcare services, though they do not yet match traditional Tier 1 cities in global influence. In comparison, Tier 2 and Tier 3 cities have fewer resources and lower economic activity, while Tier 4 and Tier 5 cities, often in rural areas, face challenges such as limited healthcare infrastructure; (2) household registration status was represented, distinguishing between urban and rural citizenship; (3) migrant residence indicated children living in a location different from their household registration or not; (4) Residence–hospital distance was measured by the distance (in km) between the household registration location and the hospital where children received their autism diagnosis. When the diagnosis occurred at the household registration location, the distance was recorded as 0. Additionally, other variables considered were (5) annual family household income; (6) misdiagnosis, capturing whether the child had experienced misdiagnosis; and (7) the number of clinicians seen before diagnosis. If the first clinician the child saw gave a diagnosis of autism, the number was recorded as 0.

Need factors reflect an individual’s perceived or evaluated need for healthcare services, such as (1) severity level at diagnosis, provided by clinicians and ranging from mild to severe. For cases where clinicians did not provide a severity level, i.e., parents indicating “Not provided”, prompted follow-up questions for them to self-identify their child’s severity at that time; (2) current severity level was measured using the Clancy Autism Behavior Scale (CABS), a standardized assessment tool designed to measure and evaluate the severity of autism symptoms and behaviors [39]; type of (3) medical comorbidities and (d) initial symptoms indicating developmental concerns observed by parents/caregivers.

### Statistical analysis

This study utilized age criteria for autism diagnosis based on prior research conducted in China [13]. Participants were divided into three age groups: the early diagnosis group (diagnosis age < 24 months), the non-early diagnosis group (diagnosis age between 24 and 36 months), and the late diagnosis group (diagnosis age > 36 months). To assess differences among these groups in the predisposing, enabling, and need factors, ANOVA for continuous variables and Chi-square tests for categorical variables were employed. Subsequently, significant variables were selected as predictors to investigate their associations with different age groups of autism diagnosis, using ordinal logistic regressions. To evaluate multicollinearity, variance inflation factor (VIF) was assessed. A VIF greater than 5 indicates a potential presence of multicollinearity in the model [40]. Statistical analyses were performed using RStudio [41], with significance set at a *p*-value of 0.05.

## Results

**Fig. 1:**
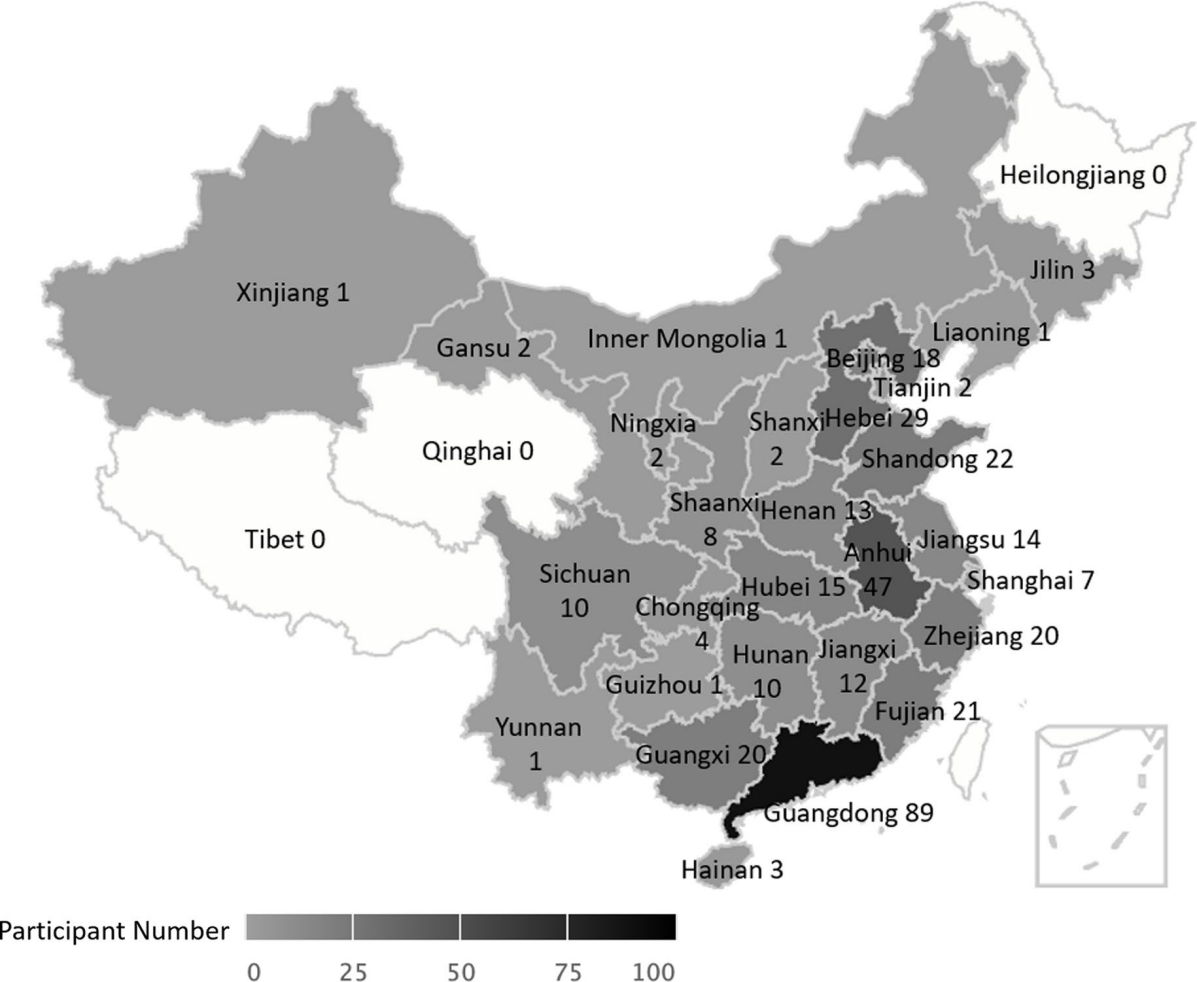
Geographical distribution of participants across Chinese Mainland. Color intensity reflects the number of participants in each province, with darker shades representing provinces with higher participant numbers. Key provinces are labeled with participant numbers

A total of 378 valid surveys were included in the data analysis, covering 90.3% of the provinces in Chinese Mainland (see Fig. 1), with the remaining provinces not represented due to challenges in participant outreach, such as geographic remoteness. The surveys predominantly emanated from parents (*n* = 360, 95.24%), with 4.76% being completed by other caregivers (*n* = 18). The mean age at which concerns about children’s development were first raised was 27.88 months (SD = 16.47). In contrast, the mean age at which autism was diagnosed was 38.81 months (SD = 19.10), resulting in a discrepancy of 10.93 months. The majority of children in our sample received their diagnosis after 24 months, with 41.27% diagnosed between 24 and 36 months, 44.97% diagnosed after 36 months, and only 13.75% diagnosed earlier than 24 months. At the time of the study, the mean age of the children was 67.09 months (SD = 33.26), with the majority being male (79.89%). Among caregivers who reported the severity level assigned by clinicians at the time of diagnosis, 24.60% were classified as mild, 23.54% as moderate, and only 11.38% as severe, while 40.84% of caregivers reported no such information provided by clinicians. According to caregivers’ self-reports, the majority of these children were at the severe level (30.78%), followed by the moderate level (25.49%), and then the mild level (13.73%). Moreover, approximately 14.81% of children experienced misdiagnosis before receiving a formal autism diagnosis, and on average, more than two clinicians were consulted before an autism diagnosis was reached (Mean = 1.41; SD = 1.55). Most parents in the study were married (87.30%) and held a bachelor’s degree or above (64.02%). Geographically, these families were evenly distributed across different city tiers, ranging from tier 1 to tier 5 (14.29-25.13%), with the majority residing in urban areas (71.01%) and holding local household registrations (81.22%). The mean annual family income was 244,000 (SD = 544,000) RMB.

Table 1 illustrates the results of the data analysis comparing the three age groups. The child’s current age emerged as the sole significant factor among the predisposing factors (F(2,375) = 23.00, *p* < 0.001, η^2^ = 0.11). In contrast, other predisposing factors, such as the child’s sex, parental highest education level, and marital status, did not exhibit significant differences across the three age groups (*p* > 0.05). Turning to the enabling factors, several variables proved to be significant. These included residence status (X^2^(2,376) = 7.84, *p* = 0.02, V = 0.14), distance of residence from the hospital (F(2,375) = 3.39, *p* = 0.04, η^2^ = 0.02), whether the child had experienced misdiagnosis (X^2^(2,378) = 24.00, *p* < 0.001, V = 0.25), and the number of clinicians consulted before receiving an autism diagnosis (F(2,375) = 3.56, *p* = 0.03, η^2^ = 0.02). As for need factors, all variables were found to be significant, except for the current severity level (F(2,375) = 2.94, *p* = 0.054, η^2^ = 0.02). The significant variables encompassed the severity level at the time of diagnosis (X^2^(4,378) = 19.57, *p* < 0.001, V = 0.16), the total number of medical comorbidities (F(2,375) = 3.23, *p* = 0.04, η^2^ = 0.02), and the initial symptoms related to developmental concerns in children (F(2,375) = 3.76, *p* = 0.02, η^2^ = 0.02).

**Table 1:** Demographic characteristics and key predictors overall and by different age groups of diagnosis

	Age of autism diagnosis						
	< 24 months (*n* = 52)	24–36 months (*n* = 156)	> 36 months (*n* = 170)	F/Χ²	*p*	Effect size	Total (*n* = 378)
**Dependent Variables**							
Age of Initial Concerns (Month)	**16.30(4.74)**	**23.40(6.93)**	**35.28(20.87)**	**41.25**	**< 0.001**	**0.19**	**27.88(16.47)**
Age of Diagnosis (Month)	**19.71(3.08)**	**29.77(3.83)**	**52.94(20.23)**	**171.1**	**< 0.001**	**0.48**	**38.81(19.10)**
**Predisposing factors**							
Child Age (Month)	**48.87(27.59)**	**60.76(30.22)**	**78.47(33.62)**	**23.00**	**< 0.001**	**0.11**	**67.09(33.26)**
Child Sex				1.20	0.55	0.06	
Female	11(21.15%)	35(22.44%)	30(17.65%)				76(20.11%)
Male	41(78.85%)	121(77.56%)	140(82.35%)				302(79.89%)
Highest Level of Parental Education				4.27	0.37	0.08	
College and Below	25(48.08%)	53(33.97%)	58(34.12%)				136(35.98%)
Bachelor	20(38.46%)	69(44.23%)	77(45.29%)				166(43.91%)
Master and Above	7(13.46%)	34(21.80%)	35(20.59%)				76(20.11%)
Marital Status				7.57	0.27	0.10	
Married	45(86.54%)	135(86.54%)	150(88.24%)				330(87.30%)
Divorced/Separated/Widowed	5(9.61%)	16(10.26%)	15(8.82%)				36(9.52%)
Never married	2(3.85%)	2(1.28%)	0(0%)				4(1.06%)
Others	0(0%)	3(1.92%)	5(2.94%)				8(2.12%)
**Enabling factors**							
Household Registration (Hukou)				7.10	0.53	0.10	
Tier 1	9(17.31%)	41(26.28%)	36(21.18%)				86(22.75%)
New Tier 1	14(26.92%)	35(22.44%)	28(16.47%)				77(20.37%)
Tier 2	10(19.23%)	26(16.67%)	30(17.65%)				66(17.46%)
Tier 3	12(23.08%)	36(22.08%)	47(27.65%)				95(25.13%)
Tier 4 and 5	7(13.46%)	18(11.54%)	29(17.06%)				54(14.29%)
Residence Status (Hukou)				**7.84**	**0.02**	**0.14**	
Urban Citizen	29(55.77%)	118(76.13%)	120(71.01%)				267(71.01%)
Rural Citizen	23(44.23%)	37(23.87%)	49(28.99%)				109(28.99%)
Migrant residence (Hukou)				2.94	0.23	0.09	
No	40(76.92%)	133(85.26%)	134(78.82%)				307(81.22%)
Yes	12(23.08%)	23(14.74%)	36(21.28%)				71(18.78%)
Misdiagnosis				**24.00**	**< 0.001**	**0.25**	
No	49(94.23%)	145(92.95%)	128(75.29%)				322(85.19%)
Yes	3(5.77%)	11(7.05%)	42(24.71%)				56(14.81%)
Residence–Hospital Distance (km)	**94.64(196.16)**	**102.52(242.84)**	**185.57(395.56)**	**3.39**	**0.04**	**0.02**	**138.78(318.34)**
Annual Family Income (100,000 RMB)	2.82(7.33)	2.06(2.36)	2.68(6.67)	0.68	0.51	0.004	2.44(5.44)
Number of Clinicians Seen Before Diagnosis	**1.46(1.72)**	**1.16(1.28)**	**1.61(1.68)**	**3.56**	**0.03**	**0.02**	**1.41(1.55)**
**Need factors**							
Severity Level at Diagnosis				**19.57**	**< 0.001**	**0.16**	
Mild	7(13.46%)	37(23.72%)	49(28.83%)				93(24.60%)
Moderate	5(9.62%)	44(28.21%)	40(23.53%)				89(23.54%)
Severe	8(15.38%)	15(9.62%)	20(11.76%)				43(11.38%)
Not provided	32(61.54%)	60(38.46%)	61(35.88%)				153(40.48%)
*For families whose severity was not provided by clinicians at the time of diagnosis*,* severity was based on caregivers’ reports*							
Mild	2(6.24%)	5(8.33%)	14(22.95%)				21(13.73%)
Moderate	7(21.88%)	12(20%)	20(32.79%)				39(25.49%)
Severe	23(71.88%)	43(71.67%)	27(44.26%)				93(30.78%)
Current Severity Level in CABS Scores	16.81(5.19)	15.03(4.84)	14.97(5.09)	2.94	0.054	0.02	15.25(5.03)
Medical Comorbidities	**0.08(0.44)**	**0.18(0.72)**	**0.34(0.81)**	**3.23**	**0.04**	**0.02**	**0.24(0.74)**
Attention-Deficit/Hyperactivity Disorder	1(1.92%)	8(5.13%)	20(11.76%)				29(7.67%)
Dyslexia	1(1.92%)	3(1.92%)	1(0.59%)				5(1.32%)
Depression	0(0%)	1(0.64%)	1(0.59%)				2(0.53%)
Anxiety Disorder	0(0%)	2(1.28%)	3(1.76%)				5(1.32%)
Epilepsy	0(0%)	2(1.28%)	1(0.59%)				3(0.79%)
Sleep Disorder	0(0%)	3(1.92%)	5(2.94%)				8(2.12%)
Gastrointestinal Problems	0(0%)	2(1.28%)	5(2.94%)				7(1.85%)
Communication Disorder	1(1.92%)	3(1.92%)	7(4.11%)				11(2.91%)
Others	1(1.92%)	4(2.56%)	14(8.24%)				19(5.03%)
Initial Symptoms Observed by Parents	**3.37(1.50)**	**3.15(1.51)**	**2.79(1.57)**	**3.76**	**0.02**	**0.02**	**3.02(1.54)**
No Eye Contact	37(71.15%)	103(66.03%)	88(51.76%)				228(60.32%)
No Response to Name	42(80.77%)	124(79.49%)	112(65.88%)				278(73.55%)
No Gestures	30(57.69%)	69(44.23%)	60(35.29%)				159(42.06%)
Language Delay	41(78.85%)	107(68.59%)	110(64.71%)				258(68.25%)
Non-functional Use of Items	12(23.08%)	33(21.15%)	35(20.59%)				80(21.16%)
Regression	10(19.23%)	36(23.08%)	42(24.71%)				88(23.28%)
Others	3(5.77%)	20(12.82%)	28(16.47%)				51(13.49%)

Continuous variables are presented as mean (standard deviation, SD) and categorical variables as number (percentage). To compare between-group differences, ANOVA F-test and Chi-Squared Test were applied for continuous and categorical variables respectively. Effect sizes for the ANOVA F-test were given by the partial eta squared and for the Chi-Squared Test, were given by Cramer’s V. Bold values indicate statistical significance at *p* < 0.05

The significant variables identified through ANOVA and Chi-square tests across different age groups underwent further analysis as potential predictors for varying age groups of autism diagnosis. Table 2 presents the results of the ordinal logistic regression, with the three age groups of diagnosis ordered chronologically as < 24 months, 24–36 months, and > 36 months. The analysis revealed that among predisposing factors, the current age of autistic children was a significant factor. Younger autistic children tended to be in the younger age group of diagnosis (β = 0.02, t = 5.91, *p* < 0.001). Specifically, for every one-month decrease in the age of autistic children, the odds of being diagnosed at a younger age increased by 1.02 times. Among enabling factors, the experience of misdiagnosis (β = 1.42, t = 3.99, *p* < 0.001), and the distance between residence and hospital (β = 0.009, t = 2.16, *p* = 0.03), were significant predictors. Children who experienced misdiagnosis tended to have 4.13 times higher odds of being diagnosed at a later age. Additionally, for every 100 km decrease in the distance between residence and hospital, the odds of being diagnosed at a younger age increased by 1.09 times. In terms of need factors, only the severity level at diagnosis exhibited a significant prediction (β = 0.44, t = 3.25, *p* = 0.001). Notably, greater symptom severity was associated with an earlier age of diagnosis, with 1.56 times increase in the odds of being diagnosed at a younger age for each unit increase in severity level.

**Table 2:** Predictive factors of the three age groups of diagnosis

	β	SE	t	*p*	95% CI		Odd Ratio	Collinearity
					Lower	Upper		VIF
**Predisposing factors**								
Child Age (Month)	**0.02**	**0.004**	**5.91**	**< 0.001**	**0.02**	**0.03**	**1.02[1.01**,**1.03]**	**1.02**
**Enabling factors**								
Residence Status *(Unban citizen ref*^*a*^*)*	-0.21	0.24	-0.88	0.38	-0.67	0.26	0.81[0.51,1.29]	1.04
Residence–Hospital Distance (100 km)	**0.09**	**0.04**	**2.21**	**0.03**	**0.01**	**0.16**	**1.09[1.01**,**1.18]**	**1.04**
Misdiagnosis *(No ref*^*a*^*)*	**1.42**	**0.35**	**3.99**	**< 0.001**	**0.72**	**2.11**	**4.13[2.10**,**8.51]**	**1.07**
Number of Clinicians Seen Before Diagnosis	-0.009	0.08	-0.12	0.91	-0.16	0.14	0.99[0.85,1.15]	1.09
**Need factors**								
Severity Level at Diagnosis *(Severe ref*^*a*^*)*	**0.44**	**0.14**	**3.25**	**0.001**	**0.18**	**0.71**	**1.56[1.19**,**2.04]**	**1.08**
Medical Comorbidities	0.21	0.16	1.31	0.19	-0.11	0.53	1.24[0.91,1.74]	1.07
Initial Symptoms Observed by Parents	-0.11	0.07	-1.55	0.12	-0.25	0.03	0.90[0.78,1.03]	1.08

SE: Standard Error. CI: Confidence Interval. The variance inflation factor (VIF) was calculated to assess multicollinearity among predictors. Bold values indicate statistical significance at *p* < 0.05

^a^The reference group

## Discussion

The present study investigated factors associated with the timely diagnosis of autism in China, utilizing Andersen’s Behavioral Model as a framework. Data were collected from across 90.3% of the provinces in Chinese Mainland, with families evenly distributed across various city tiers, ranging from tier 1 to tier 5 (14.29-25.13%). The findings revealed a significant trend: the majority of Chinese children (86.24%) did not receive a formal diagnosis until they were over 24 months old, indicating a delay in autism diagnosis in China, consistent with observations in other developing countries and globally [15]. Additionally, there was an average gap of 10.93 months between parents/caregivers’ initial concerns and a confirmed diagnosis, underscoring the need for improved diagnostic practices.

The discussion of our findings within the framework of Andersen’s Behavioral Model highlights the multifaceted nature of autism diagnosis in China, with predisposing, enabling, and need factors dynamically intertwined [15]. In particular, the age of children upon survey completion merged as a significant factor for timely diagnosis of autism, implying that children born more recently are more likely to receive a timely diagnosis. This finding aligns with previous research conducted in Western countries and other developing countries [8, 13–15, 29], highlighting a notable developmental pattern in the societal understanding and awareness of autism diagnosis globally.

Regarding enabling factors, the experience of misdiagnosis and the Residence–Hospital distance were significant predictors for delayed diagnosis. In our study, there was an average delay of 10.93 months between parents/caregivers’ initial concerns and the formal diagnosis of autism, which reflects critical gaps in the healthcare-seeking and diagnostic process. This delay can be attributed to enabling factors. Specifically, approximately 14.81% of children in our sample experienced misdiagnosis, which likely extended the diagnostic timeline. This may seem counterintuitive given the increasing societal awareness of autism. However, while greater societal awareness has contributed to earlier recognition of autism, challenges remain in ensuring accurate diagnosis, particularly in the context of differential diagnosis. Our findings align with previous research indicating a lack of autism-specific training and misconceptions among healthcare professionals in China, particularly in rural or less-developed areas, regarding autism symptoms, diagnosis, and intervention [24]. Additionally, the psychiatrist-to-child ratio was alarmingly low in the country, with fewer than 500 child psychiatrists available for 298 million children between the ages of 0 and 17 [25, 28, 42]. Lacking qualified specialists in autism diagnosis likely contributed to both the misdiagnosis and the prolonged delay between initial concerns and diagnosis. This highlights an urgent need for fully trained specialists in the field of autism to expedite the diagnostic process and alleviate the stress faced by families seeking definitive diagnoses.

Moreover, the significant impact of misdiagnosis on Chinese families can also be related to the prevalent stigma surrounding autism within Chinese society. A study conducted on a sample size of 1254 Chinese individuals revealed that approximately 38% of participants endorsed the stigmatization of autism, leading parents/caregivers to seek medical attention from departments other than psychiatry and exacerbating delays in diagnosis [22]. The stigma is often more pronounced in rural areas compared to urban regions, largely due to lower levels of autism awareness and limited access to specialized care. This results in tangible consequences, as rural children in China are diagnosed, on average, six months later than their urban counterparts [30]. These regional disparities contribute to varying experiences and delays in diagnosis, with families in rural areas facing additional challenges in accessing appropriate care. Compounding this issue is the absence of a comprehensive referral health system in China, leaving parents/caregivers without guidance on which department to visit for specialist care at hospitals [25, 28]. This reveals the urgency and the necessity of developing a structured referral system in China to guide parents/caregivers toward specialist care and reduce the influence of social stigma on healthcare-seeking behaviors.

Meanwhile, the Residence–Hospital distance can significantly predict the different age groups of autism diagnosis, with closer proximity associated with earlier diagnosis. However, in China, the availability of child psychiatrists is not only limited in number but also concentrated in high-income Tier 1 cities, such as Beijing and Shanghai [43], despite over 70% of the population residing in lower-tier cities [44, 45]. This distribution underscores a glaring disparity in China’s autism diagnosis services, where accessing highly qualified specialists necessitates substantial time and financial investment. This challenge presents a formidable obstacle for Chinese caregivers seeking timely autism diagnoses for their children. To mitigate this geographical imbalance, telehealth has emerged as a promising solution [46], especially given that autism diagnosis heavily relies on behavioral observations. Telehealth initiatives could include virtual consultations with specialists, caregiver-led behavioral assessments facilitated by remote clinicians, and video recordings for developmental monitoring and screening. For example, caregivers could record and upload videos of their child’s behaviors for review by trained specialists, using culturally validated tools for Chinese-speaking children, such as the Social Communication Scale (SCS) [47] and the Systematic Observation of Red Flags (SORF) [48]. These solutions can improve access to diagnostic services in rural or underserved areas, reducing the time, travel, and costs associated with in-person visits.

Turning to need factors, the severity of autistic symptoms predicted the age of diagnosis, as also shown in other studies [13, 20, 48]. While this finding sheds light on the diagnosis process, it also raises concerns about the potential oversight of mild and moderate cases, leading to delays in diagnosis. This underscores the importance of implementing routine screening programs in Chinese communities using culturally validated screening tools to identify Chinese children at a high likelihood for autism, including those with mild or moderate symptoms. Encouraging regular developmental monitoring during well-child visits would also be beneficial. Healthcare providers should closely track a child’s developmental milestones and flag any delays or atypical behaviors for further evaluation. Moreover, to uncover subtle signs of autism in mild and moderate cases, a multidisciplinary approach involving pediatricians, psychiatrists, speech therapists, and other specialists should be taken.

In addition, despite the significant effect of autism severity level on the age of autism diagnosis, a notable ratio of our caregivers (48.4%) reported that they did not receive the severity level of children’s autism from clinicians. Among those who received the severity report, the severity level was mostly reported as “mild” (24.6%). As the child’s autism severity correlates with parental stress, depressed mood, and perceived quality of life, coupled with traditional Chinese values emphasizing success in children, clinicians may downplay severity to avoid distressing parents/caregivers [49–53]. However, withholding or downplaying severity information can have significant consequences. Parents/caregivers who are unaware of the severity of their child’s condition may underestimate the need for early intervention and miss the critical window for timely and effective support. This raises ethical concerns about the balance between transparency and cultural sensitivity in severity reporting. On the one hand, providing an accurate severity level is essential for empowering parents/caregivers to seek appropriate interventions and resources for their child. On the other hand, openly discussing severity may cause significant emotional distress for families, particularly in the context of societal stigma surrounding autism in China, where a child’s success is often seen as a reflection of the family’s social standing. Clinicians must navigate this tension carefully, balancing the need for transparency with cultural sensitivity. This includes presenting severity information in a way that is both honest and constructive, emphasizing the availability of effective interventions and supports that can improve long-term outcomes. Policymakers should establish guidelines to support clinicians in addressing these ethical challenges. Training programs for healthcare professionals should emphasize the importance of clear and transparent communication while equipping clinicians with culturally sensitive strategies to convey severity information. For instance, clinicians could adopt a strengths-based approach, framing discussions of severity in terms of both challenges and the child’s unique abilities, to help mitigate the emotional impact on families. Such strategies could foster greater trust and collaboration between families and healthcare providers, ultimately improving outcomes for autistic children.

Strikingly, none of the socio-familial factors, including parental education, household income, or marital status, were significantly associated with timely diagnosis. This finding aligns with previous studies conducted in China [13], Latin American and Caribbean countries [15], and Israel [20], where structural barriers and systemic issues often outweigh individual socio-familial factors. However, it contrasts with findings from the U.S [19]. and Denmark [8], where higher parental education and household income have been shown to predict earlier diagnosis. These discrepancies may reflect differences in healthcare systems, resource availability, and cultural attitudes toward autism diagnosis. In China, limited access to diagnostic services and stigma may reduce the influence of socio-familial factors compared to systemic barriers.

While our study sheds light on the multifaceted access and utilization of autism diagnosis services in China, it is essential to acknowledge its limitations. First, the sample size of the current study, while distributed across various provinces, is smaller compared to other cross-region studies with sample sizes exceeding 1500 autistic individuals [15, 18]. This smaller sample size may limit the statistical power to detect significant effects and the generalizability of our findings. Future studies with larger sample sizes and employing a comprehensive framework may offer deeper insights into the overall landscape of autism diagnosis in China. Secondly, self-reported data may introduce recall bias, particularly concerning the timing and severity level provided by clinicians. This bias could lead to under- or overestimation of the delay in diagnosis or the reported severity level, potentially influencing the observed relationships between key factors and diagnostic timeliness. Future research should consider incorporating objective measures, such as medical records or clinician-reported data, to validate self-reported responses and minimize recall bias. Third, our study recruited a convenience sample, with more participants from eastern provinces of China compared to middle or western regions. This geographic skew, combined with the lack of coverage in provinces such as Tibet, Qinghai and Heilongjiang, limits the generalizability of the findings and may not fully represent the experiences of parents/caregivers across all regions in China. The absence of data from Tibet, Qinghai and Heilongjiang was due to challenges in reaching participants, stemming from the geographic remoteness and limited infrastructure in these areas. Moreover, most participants in our sample reported high socio-economic status (mean annual family income of 244,000 RMB) and high educational attainment (with the majority holding a bachelor’s degree or above). This selection bias limits the representativeness of the sample, constrains the diversity of perspectives, and reduces the generalizability of the study findings. Future research is warranted to address these limitations by recruiting larger and more diverse samples, employing probability sampling methods, and expanding the geographic representation to provide a more comprehensive understanding of autism diagnosis across China.

### Policy implications

The findings of this study highlight critical areas where policies are needed to improve autism diagnosis and management in China, particularly in underserved regions. To address geographic disparities, policymakers should prioritize the phased implementation of routine autism screening programs, as outlined in the 2022 National Health Commission protocol [54]. In rural and remote regions, screenings could be integrated into existing maternal and child health visits or school entry evaluations, leveraging community health workers trained to administer simplified tools (e.g., the Modified Checklist for Autism in Toddlers, M-CHAT) [55]. However, the efficacy of these programs in low-resource settings requires rigorous evaluation, including metrics for follow-up care accessibility. Telehealth offers a scalable solution to bridge diagnostic gaps in underrepresented regions. For example, caregiver-led telehealth assessments, where parents or local providers record behavioral observations for remote specialist review have shown promise in several locally developed [47] or culturally adapted assessments [48]. Emerging technologies, such as eye-tracking devices [56–58], portable EEG systems [58–60], and MRI analysis [61], can augment diagnostic objectivity, particularly in settings with limited specialist access. However, technology must complement specialist training. In addition to support telehealth and technology integration to enhance access and mitigate diagnostic delays, policies should also promote autism-specific training for healthcare professionals and establish national accreditation standards for autism specialists and integrate multidisciplinary diagnostic teams to further improve diagnostic accuracy. The use of telehealth and technology coupled with trained personnel will enable a timelier diagnosis in the not-too-distant future.

Moreover, the pervasive stigma surrounding autism in China hinders timely diagnosis. Policymakers should prioritize culturally tailored public education campaigns to raise awareness about autism, focusing on signs, symptoms, neurodiversity, and early supports. These efforts should target both urban and rural populations and encourage culturally sensitive, non-stigmatizing care in healthcare facilities. Additionally, policymakers should offer psychosocial support for parents to help them manage the emotional challenges of autism diagnosis and the stigma they may encounter. By addressing these multifaceted challenges, policymakers can significantly improve the accessibility, timeliness, and accuracy of autism diagnosis, ensuring that all children across China receive the timely diagnosis and support they need.

## Conclusions

By applying Andersen’s Behavioral Model as a framework, we identified predisposing, enabling, and need factors that contribute to the complex landscape of autism diagnosis in China. The findings indicate that children born more recently with more severe symptoms at diagnosis are more likely to receive a timely diagnosis. Strategies including routine screening programs, regular developmental monitoring during well-child visits, and a multidisciplinary approach are essential to identify autistic children early, especially those with mild and moderate symptoms. Healthcare access factors, encompassing experience of misdiagnosis and longer distances between residence and hospitals, can significantly delay diagnosis in China. Addressing these obstacles requires investments in specialized autism training for healthcare professionals and the enhancement of referral systems. Additionally, the expansion of telehealth solutions is essential to ensure equitable access to diagnosis services across diverse regions.

## Data Availability

The data that support this manuscript will be made available upon reasonable requests submitted to the corresponding author.
